# Comparative functional characterization of novel non-syndromic *GJB2* gene variant p.Gly45Arg and lethal syndromic variant p.Gly45Glu

**DOI:** 10.7717/peerj.2494

**Published:** 2016-10-11

**Authors:** Juan Rodriguez-Paris, Jörg Waldhaus, Jeenal A. Gordhandas, Lynn Pique, Iris Schrijver

**Affiliations:** 1 Department of Pathology, Stanford University, Stanford, CA, United States of America; 2 Department of Otolaryngology, Head and Neck Surgery, Stanford University, Stanford, CA, United States of America; 3 Department of Pediatrics, Stanford University, Stanford, CA, United States of America

**Keywords:** Hearing loss, Connexin 26, IP3, FRAP, p.Gly45Arg, *GJB2*, p.Gly45Glu

## Abstract

We characterized a novel *GJB2* missense variant, c.133G>A, p.Gly45Arg, and compared it with the only other variant at the same amino acid position of the connexin 26 protein (Cx26) reported to date: c.134G>A, p.Gly45Glu. Whereas both variants are associated with hearing loss and are dominantly inherited, p.Gly45Glu has been implicated in the rare fatal keratitis-ichthyosis-deafness (KID) syndrome, which results in cutaneous infections and septicemia with premature demise in the first year of life. In contrast, p.Gly45Arg appears to be non-syndromic. Subcellular localization experiments in transiently co-transfected HeLa cells demonstrated that Cx26-WT (wild-type) and p.Gly45Arg form gap junctions, whereas Cx26-WT with p.Gly45Glu protein does not. The substitution of a nonpolar amino acid glycine in wildtype Cx26 at position 45 with a negatively charged glutamic acid (acidic) has previously been shown to interfere with Ca^2+^ regulation of hemichannel gating and to inhibit the formation of gap junctions, resulting in cell death. The novel variant p.Gly45Arg, however, changes this glycine to a positively charged arginine (basic), resulting in the formation of dysfunctional gap junctions that selectively affect the permeation of negatively charged inositol 1,4,5-trisphosphate (IP_3_) and contribute to hearing loss. Cx26 p.Gly45Arg transfected cells, unlike cells transfected with p.Gly45Glu, thrived at physiologic Ca^2+^ concentrations, suggesting that Ca^2+^ regulation of hemichannel gating is unaffected in Cx26 p.Gly45Arg transfected cells. Thus, the two oppositely charged amino acids that replace the highly conserved uncharged glycine in p.Gly45Glu and p.Gly45Arg, respectively, produce strikingly different effects on the structure and function of the Cx26 protein.

## Introduction

In this study, we examine the mechanism by which two sequence changes in the *GJB2* gene substitute different amino acids at position 45 (p.Gly45Arg and p.Gly45Glu) and give rise to two highly different phenotypes, one lethal and syndromic and the other non-lethal and non-syndromic. The *GJB2* gene is the most commonly implicated gene in hereditary hearing loss and has been associated with both autosomal recessive (DFNB1A (OMIM #220290)) and autosomal dominant (DFNA3A (OMIM #601544)) hearing impairment. More than a hundred sequence changes have been identified in the gene and most contribute to autosomal recessive non-syndromic hearing loss (NSHL). A small subset, however, lead to syndromic hearing loss inherited in a dominant manner (http://davinci.crg.es/deafness/). *GJB2* encodes the connexin 26 (Cx26) protein, one of a family of gap junction proteins that are essential for intercellular communication. Connexin protein subunits oligomerize in a set of six to form connexons (Evans, De Vuyst & Leybaert, 2006; Saez et al., 2003). These plasma membrane hemichannels facilitate communication with the extracellular environment (Kumar & Gilula, 1996) and form intercellular gap junctions by the docking of two hemichannels from apposing cells. Thus, apposing cells can exchange ions and small cytoplasmic molecules such as metabolites and second messengers (Dbouk et al., 2009; Goodenough & Paul, 2009).

Recessive NSHL is predominantly caused by loss of function of the Cx26 protein (Sanchez & Verselis, 2014; White, 2000) resulting from nonsense and missense substitutions and frameshift mutations in *GJB2* (Sanchez & Verselis, 2014; http://davinci.crg.es/deafness/). Dominant *GJB2* related hearing loss, in contrast, is solely attributable to single amino acid substitutions. These may result in dysfunctional or absent hemichannels and/or gap junctions (White, 2000). Dominant *GJB2* variants are predominantly syndromic but can be non-syndromic, manifesting as isolated sensorineural hearing loss. Syndromic *GJB2* variants cause hearing loss with skin conditions and other ectodermal abnormalities (Gerido & White, 2004; Lai-Cheong, Arita & McGrath, 2007; Richard, 2005; Van Steensel et al., 2004). The simple loss of function of Cx26 does not affect the development or function of the epidermis, suggesting that syndromic *GJB2* variants must effect a gain or alteration of function resulting in aberrant hemichannel activity in order to cause skin disease (Lee & White, 2009; Sanchez & Verselis, 2014). Most syndromic *GJB2* mutations are located in the N-terminal and the extracellular loop 1 (E1) regions of Cx26 which are involved in hemichannel/gap junction formation and Ca^2+^ dependent gating of the hemichannels (Sanchez & Verselis, 2014; Bennett et al., 2016).

The missense variant c.134G>A (p.Gly45Glu) in *GJB2* exon 2 is associated with the keratitis-ichthyosis-deafness (KID) syndrome, which is often fatal in the first year of life because of cutaneous infections and septicemia (Janecke et al., 2005). The glycine at position 45 is a highly conserved amino acid across all major connexins (Zhang & Hao, 2013) and its location in the parahelix region (residues 42–50) near the highly conserved Ca^2+^ binding amino acids p.Gly45, p.Asp46 and p.Glu47 makes p.Gly45 important in the voltage-dependent loop-gate permeability barrier in Cx26 (Kwon, Tang & Bargiello, 2013; Zonta et al., 2014; Bennett et al., 2016). Interestingly, in the Japanese population p.Gly45Glu is usually associated with p.Tyr136Stop (c.408C>A) in *cis* (p.Gly45Glu with p.Tyr136Stop). This second variant truncates Cx26 and effectively cancels the lethal effects of the p.Gly45Glu variant. Together, the two variants in *cis* represent a recessive allele (Ogawa et al., 2014).

Here, we report the identification of a single copy of the novel *GJB2* missense variant c.133G>A (p.Gly45Arg) in an individual with a likely dominantly inherited NSHL. However, unlike the fatal syndromic p.Gly45Glu variant, p.Gly45Arg at the same amino acid position presents with a non-syndromic phenotype. In order to elucidate the causes for this difference, we performed comparative computational predictions, expression studies, and functional analyses *in vitro*.

### Materials and Methods

#### Study samples

Individuals with hearing loss received *GJB2* (GenBank ID: NG_008358.1) genotyping, performed by the clinical diagnostic Stanford Molecular Pathology laboratory, as part of routine medical care. Genomic DNA was isolated from peripheral blood according to standard procedures. The second exon of the *GJB2* gene, containing the entire coding sequence, was PCR amplified and products were purified using either the Qiaquick PCR Purification Kit (Qiagen, Valencia, CA, USA) or Exo-SAP-IT (GE Healthcare Amersham, Pittsburgh, PA, USA) according to the respective protocols. Purified products were then sequenced with fluorescent di-deoxy terminators (Life Technologies, Grand Island, NY, USA) and electrophoresed on a 3130xl genetic analyzer (Life Technologies, Grand Island, NY, USA). Mutation Surveyor DNA Variant Analysis software (SoftGenetics, State College, PA, USA) facilitated the evaluation of sequence quality and the detection of sequence changes.

Two samples were selected for this study under an approved Stanford IRB protocol. Written consent was not required for this left-over DNA as per Stanford IRB approved protocol (protocol 8353, assurance number FWA00000935 (SU)). One sample from a Eurasian individual carried the novel c.133G>A, p.Gly45Arg variant. Because of incomplete family history and limited clinical testing of affected individuals, the pattern of inheritance, while most likely dominant, could not be established with certainty. The proband had post-lingual development of, thus far, mild hearing loss. A second sample from a patient with Japanese ancestry carried two variants, c.134G>A (p.Gly45Glu) and c.408C>A (p.Tyr136Stop).

#### Bioinformatic pathogenicity predictions of *GJB2* variant p.Gly45Arg

The functional effects of the novel c.133G>A, p.Gly45Arg variant were predicted using the Sorting Intolerant from Tolerant (SIFT) program (http://sift.jcvi.org), Protein Analysis Through Evolutionary Relationships (PANTHER) (http://www.pantherdb.org; Thomas et al., 2003), cSNP scoring tool, Polymorphism Phenotyping v2 (PolyPhen-2) (http://genetics.bwh.harvard.edu/pph2; Adzhubei et al., 2010) and Functional Analysis Through Hidden Markov Models (FATHMM) (http://fathmm.biocompute.org.uk/index.html; Shihab et al., 2013). Additionally, we used Mutation Prediction (MuPred) (http://mutpred.mutdb.org; Li et al., 2009) and Predictor of Human Deleterious Single Nucleotide Polymorphisms (PhD-SNP) (http://snps.biofold.org/phd-snp/phd-snp.html; Capriotti, Calabrese & Casadio, 2006) to assess predicted degree of pathogenicity. The basis on which these algorithms could be applied and the use of these algorithms to analyze *GJB2* gene missense mutations including p.Gly45Glu were previously described (Yilmaz, 2015).

#### Cloning and determination of allele configuration

Residual DNA samples from the two study subjects were used to clone the whole *GJB2* coding region from wild-type genomic sequence and the proband sequences. The sequence containing c.133G>A, p.Gly45Arg was cloned into the pDrive vector (Qiagen, Valencia, CA, USA) using the following primers: Cx26 forward: 5′-CCC*CTCGAG*AGATGG ATTGGGGCACGCTGCAGACGATC-3′and Cx26 reverse: CCC*GGATCCC*GGTAGGTCC ACCACAGGGAGCCTTCGATGCG. XboI and BamHI restriction sites were introduced into the primers for further subcloning and are shown underscored in italics. The coding sequence of the individual carrying the c.134G>A, p.Gly45Glu and c.408C>A, p.Tyr136Stop variants was also cloned using these primers in order to determine the allele configuration of the two substitutions. When it was determined that the two variants were in *cis* configuration, the sequence was cloned in the same fashion using a different reverse primer, 5′-CCC*GGATCC*GAAACTGGCTTTTTTGACTTCCCAGAACA-3′, which substituted a Tyr amino acid for the Stop codon at position 136. Clones were selected using 50 µg/mL Ampicillin (Thermo Fisher Scientific, Carlsbad, CA, USA) and miniplasmid preparations and restriction enzyme analyses were performed to identify positive clones. These were then sequenced using Sanger sequencing with fluorescent di-deoxy terminators and electrophoresed on a 3730xl genetic analyzer (Life Technologies, Grand Island, NY, USA).

#### Plasmid construction

The coding regions of the wild-type *GJB2* sequence, the p.Gly45Arg (G45R) variant sequence and the truncated sequence representing the p.Gly45Glu (G45E) and p.Tyr136Stop (Y136X, thus named because the protein is truncated at codon 136, and X is a one-letter abbreviation for a Stop codon) double variants in *cis* (G45R/Y136X and G45E/Y136X) were sub-cloned from the pDrive clones into the XhoI and BamHI sites of the pAcGFP1-Hyg-N1 and the pDsRed-Monomer-N1 vectors (Clontech, Mountain View, CA, USA). The fluorescent proteins in these and in the subsequently described constructs were located at the C-terminus of the Cx26 protein. p.Gly45Glu and p.Tyr136Stop were created separately using the QuikChange II Site-Directed Mutagenesis Kit (Stratagene, La Jolla, CA, USA). The following primers were used for PCR amplification: p.Gly45Glu forward: 5′-AAAGGAGGTGTGGGAAGATGAGCAGGCCG-3′; p.Gly45Glu reverse: 3′-TTTCCTCCACACCCTTCTACTCGTCCGGC-5′; p.Glu45Gly forward: 5′-CTCCCTGTGGTGGACCTAAACAAGCAGCATCTT-3′ and p.Glu45Gly reverse: 3′-GAGGGACACCACCTGGATTTGTTCGTCGTAGAA-5′. The mutated bases are underscored in the primer sequences and correspond to the following: G>A at *GJB2* coding nucleotide 134 resulting in Gly>Glu at codon 45 in the *GJB2*-WT-pAcGFP1-Hyg-N1 and *GJB2*-WT-pDsRed-Monomer-N1 constructs and A>G at the same nucleotide in the *GJB2*-G45E/Y136X-pAcGFP1-Hyg-N1 and *GJB2*-G45E/Y136X-pDsRed-Monomer-N1 constructs, resulting in a reversion of the Glu variant to wild-type Gly at codon 45. Positive pAcGFP1-Hyg-N1 clones were selected using 50 µg/mL ampicillin (Thermo Fisher Scientific, Carlsbad, CA, USA) and positive pDsRed-Monomer-N1 clones using 50 µg/mL kanamycin (Thermo Fisher Scientific, Carlsbad, CA, USA). Miniplasmid preparations and restriction enzyme analyses were performed to identify positive clones. All positive clones were then sequenced to verify the presence of the introduced changes and to ensure that the PCR amplification had not introduced erroneous sequence changes.

To create the fusion proteins of Cx26 and tags V5 and FLAG, adaptor-duplexes with the specific tag sequences flanked with 5′BamHI and 3′BsrGI restriction sites were synthesized, and subsequently subcloned into the 5′BamHI and 3′BsrGI restriction sites of the *GJB2*-pAcGFP1-Hyg-N1 constructs, replacing the GFP1 sequence with that of the epitope tag (Zhang, Scherer & Yum, 2011).

Constructs *GJB*2-pcDNA3.1(-)-EGFP and *GJB2*-pcDNA3.1(-)-mRFP, which were generously provided by Drs. Ogawa and Akiyama (Ogawa et al., 2014), were used to generate the *GJB*2-G45R-pcDNA3.1(-)-EGFP, *GJB2*-G45R-pcDNA3.1(-)-mRFP, *GJB*2-Y136X-pcDNA3.1(-)-EGFP and *GJB2*-Y136X-pcDNA3.1(-)-mRFP constructs by site directed mutagenesis as described above. The following primers were used for PCR amplification: Gly45Arg forward: 5′-CAAAGGAGGTGTGGAGAGATGAGCAGGCC-3′; Gly45Arg reverse: 3′-GGCCTGCTCATCTCTCCACACCTCCTTTG-5′; Glu45Gly forward: 5′-AAAGG AGGTGTGGGGAGATGAGCAGGCCG-3′ and Glu45Gly reverse: 3′-CGGCCTGCTCAT CTCCCCACACCTCCTTT-5′, respectively. The mutated bases are underscored in the primer sequences and correspond to the following: G>A at *GJB2* coding nucleotide 133 resulting in Gly>Arg at codon 45 of constructs *GJB*2-pcDNA3.1(-)-EGFP and *GJB2*-pcDNA3.1(-)-mRFP and A>G at coding nucleotide 134 resulting in Glu>Gly at codon 45 of constructs *GJB*2-G45E/Y136X-pcDNA3.1(-)-EGFP and *GJB2*-G45E/Y136X-pcDNA3.1(-)-mRFP.

#### Transient transfection and fluorescence microscopy

HeLa cells, which are devoid of Cx26 expression, were purchased from American Type Culture Collection (Manassas, VA, USA) and were cultured according to standard procedures in Eagle’s Minimum Medium (EMEM) supplemented with 10% fetal bovine serum and 100U/100 µg/mL penicillin/streptomycin (Thermo Fisher Scientific, Carlsbad, CA, USA). Twenty-four hours before transfection, the HeLa cells were trypsinized and plated into a six-well plate with 18 × 18 mm^2^ glass cover-slips in each well and containing medium without antibiotics. The transfection was performed using 5 µg of the expression vectors described above and 10 µL of Lipofectamine 2000 reagent (Thermo Fisher Scientific, Carlsbad, CA, USA) per well, according to the manufacturer’s instructions. The transfections with different constructs (wild-type, p.Gly45Arg, p.Gly45Glu/p.Tyr136Stop, p.Gly45Glu and p.Tyr136Stop) were performed in the following combinations: *cis*, *trans*, heterozygous and homozygous. The p.Gly45Glu and p.Tyr136Stop variants were co-transfected in both *cis* and *trans* as a control in order to confirm that the truncating variant reverses the deleterious effects of p.Gly45Glu in *cis*. The expression of the Cx26 constructs was analyzed 48 h after transfection. HeLa cells were fixed for 10 min at room temperature with 4% paraformaldehyde in phosphate buffered saline (PBS) and permeabilized with 0.2% Triton X-100 (Sigma, St. Louis, MO, USA) after washing with PBS. Coverslips were mounted with Prolong Gold antifade reagent with DAPI stain (Thermo Fisher Scientific, Carlsbad, CA, USA) on glass slides and the fluorescence was visualized and photographed using the confocal laser-scanning microscope Zeiss LSM 700 (Zeiss United States, Pleasanton, CA, USA).

#### Inositol trisphosphate (IP_3_)-uncaging with calcium imaging and fluorescence recovery after photo bleaching (FRAP) assays

Live cells were transfected, grown on coverslips and mounted using a confocal imaging chamber (Warner Instruments, Hamden, CT, USA). After initial equilibration with Hanks’ balanced salt solution (HBSS) (Life Technologies, Grand Island, NY, USA), cells were loaded for 60 min at room temperature with 1 µM caged-IP_3_ (Enzo Life Sciences, Inc., Farmingdale, NY, USA), 5 µM Rhod2-AM (Life Technologies, Grand Island, NY, USA), 1 µM Calcein Violet-AM (Life Technologies, Grand Island, NY, USA), 250 µM sulphinpyrazone, and 0.01% w/v pluronic F-127 to prevent dye sequestration and secretion. We imaged the cells after washing for 30 min in 250 µM sulphinpyrazone/Optimem (Life Technologies, Grand Island, NY, USA) using a 20× air objective mounted on a confocal microscope (LSM700; Zeiss United States, Pleasanton, CA, USA. The parallel loading of caged IP3, Calcium sensor and the FRAP dye in combination with their spectral profiles allows for simultaneous imaging of IP3-coupling and FRAP, because both protocols use the 405 nm laser line for photo activation and photo bleaching, respectively. In this experiment we temporally separated both imaging protocols in two sections (IP3 and FRAP), both starting with identical laser irradiation steps, because repeated scanning with the 405 nm laser line, which is necessary to record Calcein Violet recovery, may result in random IP3-uncaging events in cells next to the intentionally irradiated cell.

Single Connexin26-EGFP positive cells were subjected to two consecutive focal irradiation intervals at 405 nm for a duration of 7.5 ± 3.1 s (mean ± SD, *n* = 12) depending on cell size. After the first irradiation interval we tested for IP_3_-coupling by monitoring relative calcium concentrations reflected by Rhod2-AM fluorescence intensity levels (excitation: 555 nm, emission filter: LP 560 nm) for 1 min in one second intervals. IP_3_ coupling was assessed by measuring Rhod2-AM fluorescence intensity levels in the uncaged cell and the cell directly coupled with the irradiated cell.

The second laser irradiation was initiated 3 min after the first interval and the same Connexin26-EGFP expressing cell was focally irradiated a second time using the same parameters as described earlier. Microscope detector settings were changed to imaging for 10 min in one second intervals using an excitation of 405 nm and an emission filter of SP 490 nm to record Calcein Violet fluorescence intensity levels after photo bleaching. Using these parameters the target cell was bleached to at least 50% fluorescence intensity levels compared to the time point before laser irradiation. For dye coupling analysis, we determined fluorescence intensity levels within the bleached (b) area and a control unbleached (u) area. We calculated ratios of fluorescence intensities (fb/fu) for the complete time course. Dye recovery of more than 100% relative fluorescence intensity relates to the consecutive IP3/FRAP assay with two irradiation steps. FRAP was recorded after the second irradiation step, 3 min after the first laser pulse, which is why Calcein violet may recover within the next 10 min beyond intensity levels measured before the second pulse eventually. Gap junctions were blocked by washing cells with 100 µM carbenoxolone/Optimem (Life Technologies, Grand Island, NY, USA).

## Results

### Comparative pathogenicity predictions of Cx26 variants p.Gly45Arg and p.Gly45Glu

The pathogenic effects of the novel missense variant c.133G>A, p.Gly45Arg on the *GJB2* protein were estimated using four bioinformatics programs that use different prediction algorithms: SIFT (http://sift.jcvi.org), PANTHER (http://www.pantherdb.org; Thomas et al., 2003), PolyPhen-2 (http://genetics.bwh.harvard.edu/pph2; Adzhubei et al., 2010) and FATHMM (http://fathmm.biocompute.org.uk/index.html; Shihab et al., 2013). We then compared the predicted results with those for the c.134G>A, p.Gly45Glu variant. The SIFT, PolyPhen-2, FATHMM, and PANTHER programs predicted both variants to be damaging/deleterious with a high degree of confidence (Table 1). The PhD-SNP tool (http://snps.biofold.org/phd-snp/phd-snp.html; Capriotti, Calabrese & Casadio, 2006), a predictor of the pathogenicity of single nucleotide polymorphisms, determined that both variants were disease-associated (Table 1). The MuPred server (http://mutpred.mutdb.org; Li et al., 2009), an algorithm used to predict effects on protein secondary structure, predicted a “gain of solvent accessibility” for both variants; however, the effect for the c.134G>A, p.Gly45Glu variant was predicted as a “confident hypothesis,” whereas p.Gly45Arg was predicted as an “actionable hypothesis” (Table 1).

**Table 1:** Protein prediction analyses of the Cx26 variants p.Gly45Arg and p.Gly45Glu.

Protein prediction algorithm	*GJB2* c.133G>A, p.Gly45Arg	*GJB2* c.134G>A, p.Gly45Glu
SIFT	Damaging	Damaging
PolyPhen-2	Damaging	Damaging
PANTHER	Deleterious	Deleterious
FATHMM	Damaging	Damaging
PhD-SNP	Disease	Disease
MuPred	Gain of solvent accessibility	Gain of solvent accessibility

### Expression of Cx26 variants in HeLa cells

**Figure 1:**
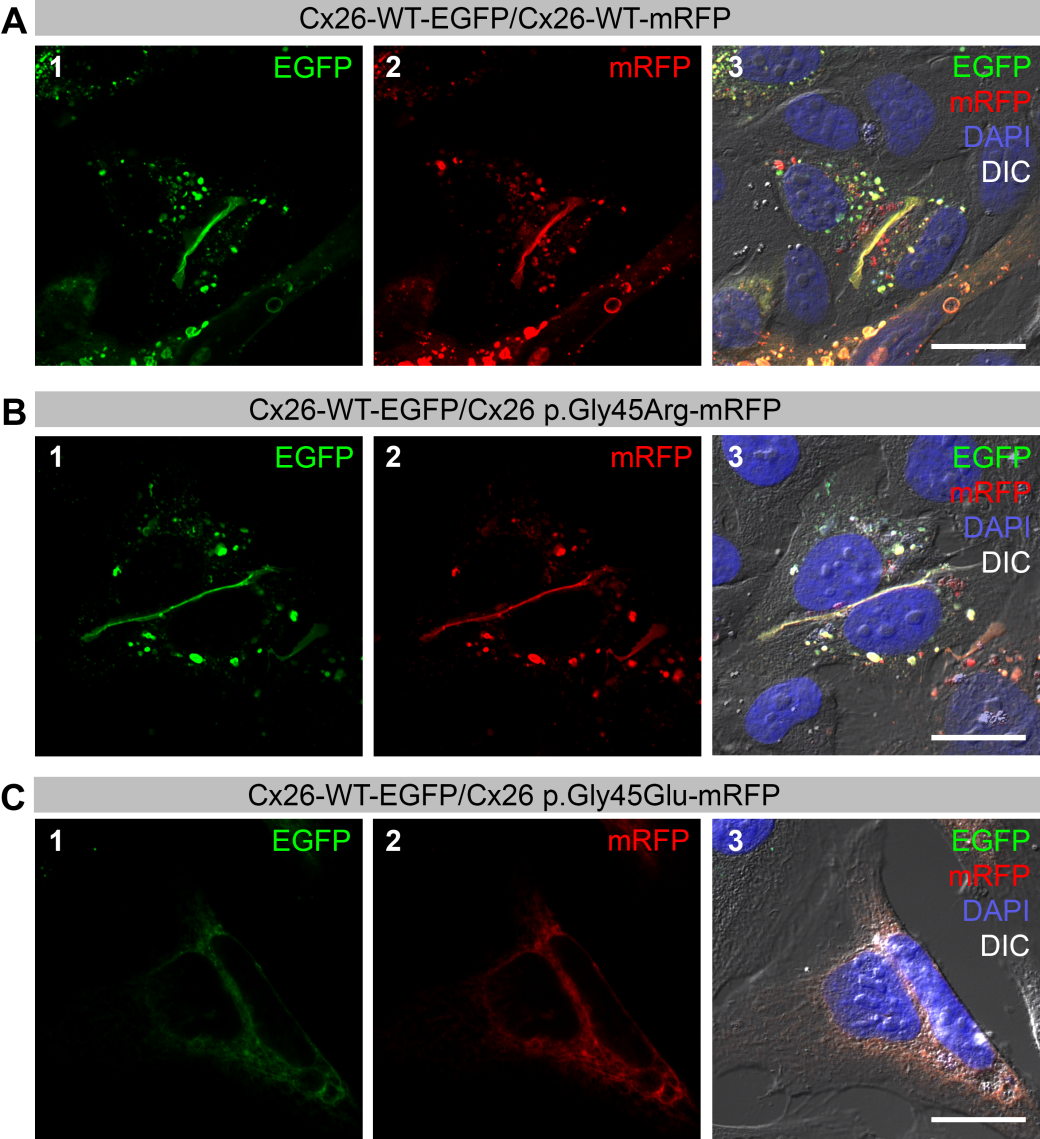
Co-expression of Wild-type (WT) Cx26 with Cx26 variants in HeLa cells. Green color corresponds to Enhanced Green Fluorescent Protein (EGFP) and red color corresponds to monomeric Red Fluorescent Protein (mRFP). Yellow color indicates areas of co-localization. DAPI staining is represented by the blue color. (A) Confocal images of transiently co-transfected HeLa cells that express Cx26-WT with C-terminal EGFP-tag (panel 1) and Cx26-WT with C-terminal mRFP-tag (panel 2). Co-localization of both proteins at the gap junctions is shown in yellow (panel 3). (B) Co-expression of Cx26-WT-EGFP (panel 1) and Cx26-p.Gly45Arg with C-terminal tagged mRFP (panel 2) results in co-localization of both proteins at the gap junctions (panel 3). (C) Cells co-transfected with Cx26-WT-EGFP (panel 1) and Cx26-p.Gly45Glu-mRFP (panel 2) do not form gap junctions as evidenced by the absence of plaques (panel 3). Scale bars: 20 µm.

The subcellular localization of differentially tagged Cx26 protein variants was determined in transiently transfected HeLa cells. Cx26-WT-EGFP (Fig. 1A, panel 1) and Cx26-WT-mRFP (Fig. 1A, panel 2) proteins were co-localized on the cell membrane and formed gap junctions as indicated by the characteristic plaques between two adjacent cells (Fig. 1A, panel 3). Subcellular co-localization was also found in cells co-transfected with Cx26-WT-EGFP and Cx-26 p.Gly45Arg-mRFP (Fig. 1B, panels 1, 2 and 3). Conversely, co-transfected Cx26-WT-EGFP and Cx26 p.Gly45Glu proteins failed to form gap junctions and cells started dying 24 h after transfection (Fig. 1C, panels 1, 2 and 3). Hence, the negatively charged amino acid at position 45 in the p.Gly45Glu variant produces a dramatically different result than the positively charged arginine at the same position in the novel p.Gly45Arg variant.

**Figure 2:**
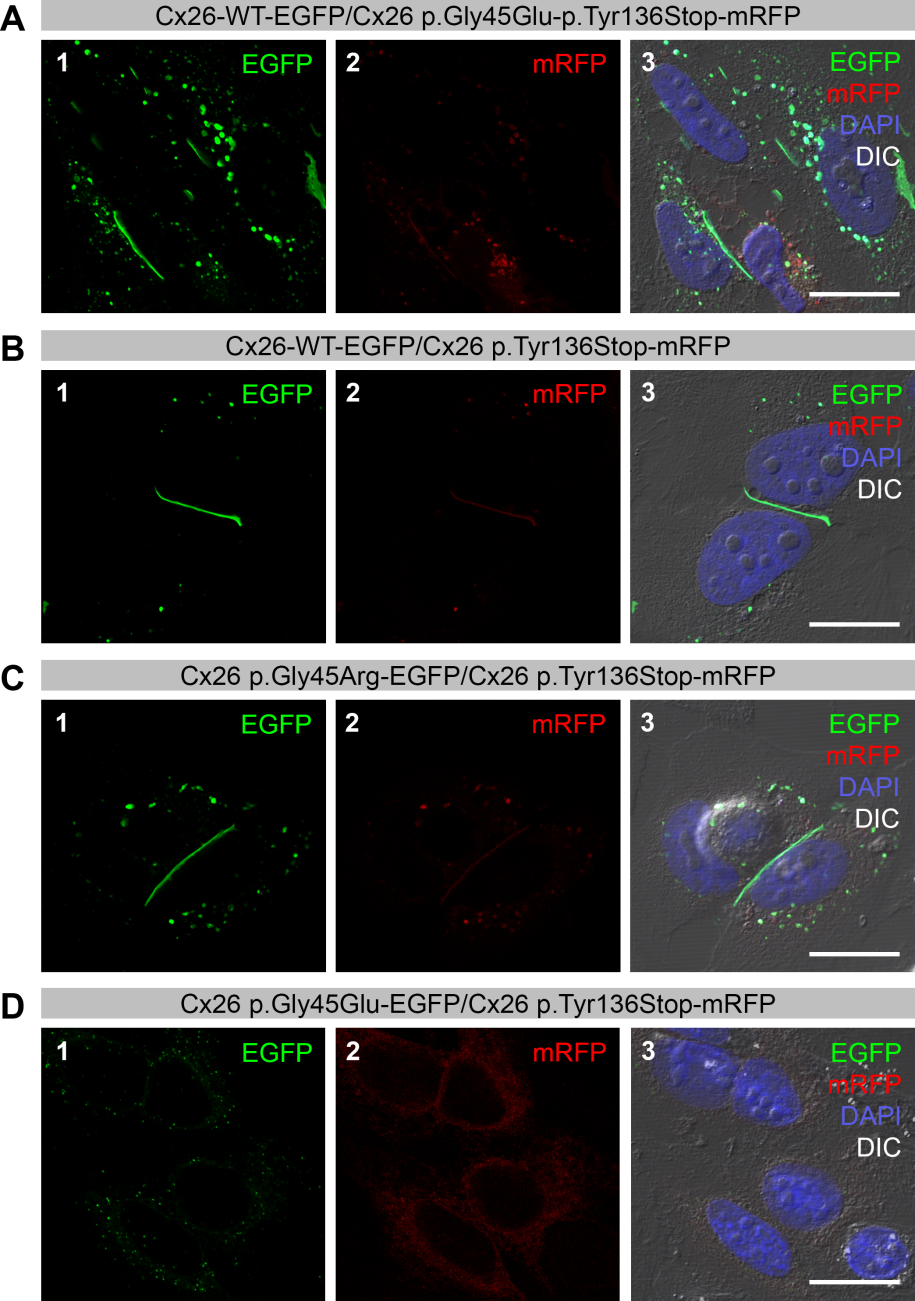
Co-expression of Cx26 variants with p.Tyr136Stop in HeLa cells. Green color corresponds to Enhanced Green Fluorescent Protein (EGFP) and red color corresponds to monomeric Red Fluorescent Protein (mRFP). Yellow color indicates areas of co-localization. DAPI staining is represented by the blue color. (A–B) Cells co-transfected with Cx26-WT-EGFP (panel 1) and, respectively, Cx26 p.Gly45Glu-p.Tyr136Stop-mRFP (panel 2) (A) and Cx26 p.Tyr136Stop-mRFP (panel 2) (B) form only Cx26-WT gap junctions (green color; panels 1 and 3).(C) Cells co-transfected with Cx26 p.Gly45Arg-EGFP (panel 1) and Cx26 p.Tyr136Stop-mRFP (panel 2) in *trans* only form Cx26 p.Gly45Arg gap junctions (green color; panels 1 and 3). Brightness and contrast were adjusted to show fluorescent signal indicating successful transfection of the cells. (D) Cells co-transfected with Cx26 p.Gly45Glu-EGFP (panel 1) and Cx26 p.Tyr136Stop-mRFP (panel 2) in *trans* do not form gap junctions (panels 1, 2 and 3). Brightness and contrast were adjusted to show fluorescent signal indicating successful transfection of the cells. Scale bars: 20 µm.

We confirmed that, as previously reported (Ogawa et al., 2014), the Cx26 p.Tyr136Stop variant in *cis* with p.Gly45Glu creates a loss of function allele that nullifies the effects of the latter variant on wild-type protein localization and leaves the ability to form gap junctions intact (Fig. 2A, panels 1, 2 and 3). Cx26 p.Tyr136Stop protein in *trans* does not have an effect on either the localization or the ability to form gap junctions with either the Cx26-WT (Fig. 2B, panels 1, 2 and 3) or the Cx26 p.Gly45Arg variant (Fig. 2C, panels 1, 2 and 3). As expected, cells co-transfected with the Cx26 p.Gly45Glu and the Cx26 p.Tyr136Stop proteins do not form gap junctions and start dying 24 h after transfection (Fig. 2D, panels 1, 2 and 3), behaving similarly to cells co-transfected with p.Gly45Glu and the Cx26-WT protein (Fig. 1C). As a control, we repeated all transfections, switching the tagged fluorescence proteins in the constructs, and obtained the same results (Data S1).

### IP_3_ permeability and fluorescence recovery after photo-bleaching (FRAP) in Cx26-G45R coupled cells

**Figure 3:**
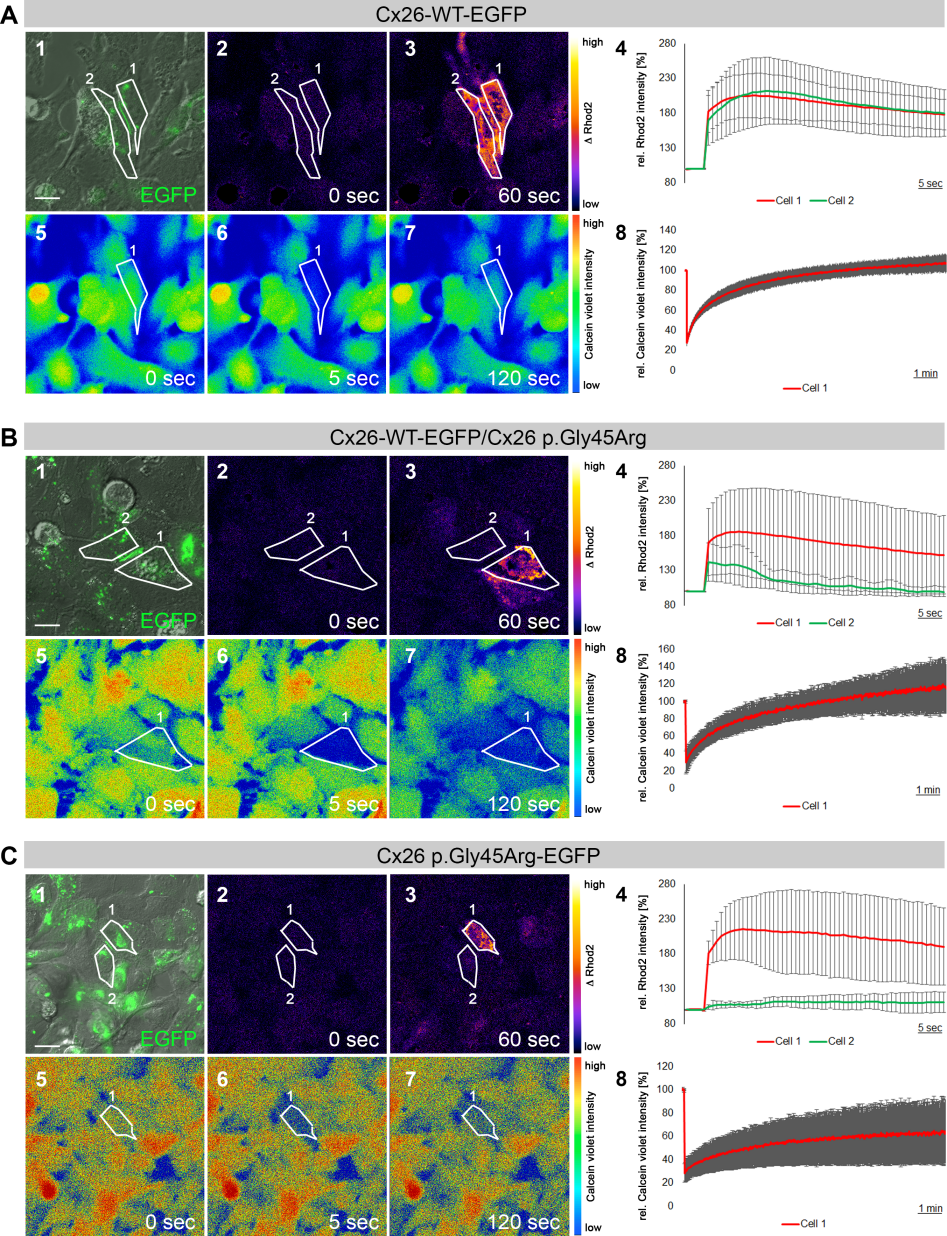
IP_3_-permeability and fluorescence recovery after photo bleaching (FRAP) in Cx26-G45R coupled cells. (A) Cx26-WT-EGFP transfected HeLa cells. Cell 1 and cell 2 are connected by gap junctions expressing Cx26-WT-EGFP (panel 1). Cells are loaded with caged IP_3_, Rhod2 AM and Calcein violet AM. Upon focal irradiation of cell 1, functional IP_3_ permeability allows for increase in Calcium levels in cell 1 and 2 as measured by changes in relative Rhod2 fluorescence intensity levels (*n* = 4; mean ± SD) (panels 2, 3 and 4). Consecutive photo-bleaching of Calcein violet in cell 1 reduces detected fluorescence levels immediately after bleaching (panels 5, 6 and 8). Calcein violet dye flow through the gap junction recovers fluorescence values (*n* = 4; mean ± SD) (panels 7 and 8). (B) Cx26-WT-EGFP and Cx26-G45R co-transfected HeLa cells. Cell 1 and cell 2 are connected by gap junctions co-expressing Cx26-WT-EGFP and Cx26-G45R (panel 1). Cells are loaded with caged IP_3_, Rhod2 AM and Calcein violet AM. Upon focal irradiation of cell 1, impaired IP_3_ permeability results in the reduced release of Calcium in cell 2 in comparison to cell 1 (*n* = 4; mean ± SD) (panels 2, 3 and 4). Consecutive photo-bleaching of Calcein violet in cell 1 reduces detected fluorescence levels immediately after bleaching (panels 5, 6 and 8). Calcein violet dye flow through the Cx26-WT-EGFP/Cx26-G45R gap junction recovers fluorescence values (*n* = 3; mean ± SD) (panels 7 and 8). (C) Cx26-G45R-EGFP transfected HeLa cells. Cell 1 and cell 2 are connected by gap junctions expressing Cx26-G45R-EGFP (panel 1). Cells are loaded with caged IP_3_, Rhod2 AM and Calcein violet AM. Upon focal irradiation of cell 1, loss of IP_3_ permeability results in IP3-uncoupling of cell 1 and 2 as demonstrated by the absence of increased Rhod2 fluorescence levels in cell 2 (*n* = 4; mean ± SD) (panels 2, 3 and 4). Consecutive photo-bleaching of Calcein violet in cell 1 reduces detected fluorescence levels immediately after bleaching (panels 5, 6 and 8). Calcein violet dye flow through the Cx26-G45R-EGFP gap junction recovers fluorescence values (*n* = 3; mean ± SD) (panels 7 and 8). Scale bars: 10 µm.

Since gap junctions constituted by the Cx26 p.Gly45Arg variant are properly localized to the membrane and the variant is associated with hearing loss, we hypothesized that the permeability of Cx26 to second messengers like inositol trisphosphate (IP_3_) might be affected, as previously observed in studies of the Cx26 p.Val84Leu variant (Beltramello et al., 2005). To test this hypothesis, and to assess the functionality of the gap junctions, Cx26-WT-EGFP transfected HeLa cells were loaded with caged-IP_3_, the calcium sensor Rhod2 AM, and Calcein violet. We identified pairs of cells connected by gap junctions as visualized by expression of the fluorescent EGFP-tag (Fig. 3A, panel 1). IP_3_ was uncaged by focal irradiation in only one of two coupled cells (Fig. 3A, panel 2, cell 1), whereupon IP_3_ binding to the inositol trisphosphate receptor (IP_3_R) at the endoplasmic reticulum (ER) triggered calcium release into the cytosol of the cell. Increase in free intracellular calcium resulted in higher Rhod2 fluorescence intensity levels compared to the time point before irradiation (Fig. 3A, panels 3 and 4). Because WT Cx26 is permeable for IP_3_, the second messenger diffused into the neighboring cell and triggered a similar release of calcium from the ER, as indicated by increased Rhod2 fluorescence intensity levels. In addition to investigating IP_3_ permeability, gap junction functionality was assessed by fluorescence recovery after photo-bleaching (FRAP). We bleached Calcein violet in the same cells previously irradiated to uncage the IP_3_. Photo-bleaching in Cx26-WT-EGFP transfected cells resulted in transient loss of Calcein violet fluorescence within the bleached cell (Fig. 3A, panels 5, 6 and 8) and consecutive recovery of the fluorescent signal by dye flow through the gap junction (Fig. 3A, panels 7 and 8). Fluorescence recovery was not detected in un-transfected cells or after transfected cells were exposed to the gap junction blocker carbenoxolone (Data S1). Co-transfection of Cx26-WT-EGFP and Cx26 p.Gly45Arg resulted in reduced IP_3_ permeability compared to Cx26-WT-EGFP alone (Fig. 3B, panels 1–4). Dye coupling was comparable to wild type Connexin 26 gap junctions (Fig. 3B, panels 5–8). Cells expressing only Cx26 p.Gly45Arg-EGFP were not competent to propagate the IP_3_ signal to the neighboring cell (Fig. 3C, panels 1–4), however cells showed reduced dye recovery compared to wild type Cx26, but were still functionally coupled by gap junctions as indicated by detectable FRAP (Fig. 3C, panels 5–8).

## Discussion

Glycine at amino acid position 45 is highly conserved across all major connexins in most species (Zhang & Hao, 2013). Only one variant, p.Gly45Glu, has been previously described at this amino acid position. It is associated with fatal keratitis-ichthyosis-deafness (KID) syndrome (Janecke et al., 2005) and has been observed as both a dominant *de novo* mutation (Griffith et al., 2006; Janecke et al., 2005) and a mutation inherited from one parent with germline mosaicism (Jonard et al., 2008). The fatal clinical course that typically unfolds during the first year of life is a consequence of cutaneous infections and septicemia (Janecke et al., 2005). In Japanese individuals, the p.Gly45Glu variant is usually present with p.Tyr136Stop (c.408C>A) in *cis*. Because p.Tyr136Stop truncates Cx26 and cancels the lethal effects of the p.Gly45Glu mutation, the combination of these two variants reflects a recessive mutation (Ogawa et al., 2014). Our results confirmed this observation (Fig. 2A), as the Cx26-WT allele was able to form normal gap junctions unlike the Cx26-G45E allele which, when co-expressed with Cx26-WT, is unable to form gap junctions.

Unlike p.Gly45Glu, novel variant p.Gly45Arg, while at the same amino acid position, was not associated with a syndrome although it also appeared to follow a dominant pattern of inheritance.

*GJB2* variants can be classified into two categories based upon whether or not the expressed protein reaches the cell membrane. Altered connexins resulting from the first variant category, although transported to the cell membrane, form dysfunctional gap junctions. Variants in the second category produce connexins that are not transported properly, resulting in a lack of gap junction formation (Ambrosi et al., 2013). The p.Gly45Arg variant appeared to belong to the first category given that the Cx26 protein that expressed the variant was targeted to the cell membrane where it formed gap junctions with the wild-type Cx26 protein (Fig. 1A). However, in contrast to what was expected for this category, this variant seemed not to disrupt passive ionic and/or biochemical intercellular communication (Fig. 3). Instead it selectively disrupted the permeation of inositol 1,4,5-triphosphate (IP_3_) (Fig. 3) in a manner comparable to the p.Val84Leu variant, which likely reflects the reason for the development of hearing loss (Beltramello et al., 2005). Since no rise in free Calcium concentration was observed in cells not directly coupled to the irradiated cell, we concluded that paracrine cell–cell communication via ATP did not contribute to our experimental findings. Instead our findings suggest that the increase in Calcium signal in the neighboring cell directly depended on the presence of coupling gap junctions that allow for IP_3_ signal propagation. Kinetics of Calcium signal propagation between the irradiated cell and its neighbor may be indicative for IP_3_- or paracrine signal propagation mechanisms. However, laser irradiation intervals of about 7 s depending on cell size did not allow us to access the process at a high temporal resolution.

p.Gly45Glu has been demonstrated to belong to the second variant category (Ogawa et al., 2014), resulting in abnormal protein trafficking to the cell membrane, dysfunctional hemichannels, and the lack of gap junctions in the presence of wild-type protein (Fig. 1C). Although all four protein pathogenicity algorithms (SIFT, PANTHER, PolyPhen-2 and FATHMM) as well as the PhD-SNP and MuPred bioinformatics programs (Table 1) predicted a functional effect for both Cx26 variants, the observed *in vitro* effects were drastically different.

Most syndromic *GJB2* variants located in the N-terminal and the E1 regions of Cx26 do not form gap junctions and cause impairment of hemichannel Ca^2+^ regulation (Sanchez & Verselis, 2014). Calcium is a central regulator of keratinocyte differentiation, thus the disruption of calcium hemostasis often results in KID (Elsholz et al., 2014). The p.Gly45 amino acid is located in the first Cx26 extracellular loop (E1) adjacent to p.Glu47 where they are two of the three aminoacids involved in the Ca^2+^ binding site for Cx26 gap junction hemichannels (Bennett et al., 2016; Kwon, Tang & Bargiello, 2013; Zonta et al., 2014). The p.Gly45Glu variant substitutes a negatively charged (acidic) amino acid for a nonpolar one, affecting its localization and interfering with Ca^2+^ regulation of hemichannel gating (Sánchez et al., 2010). Cells transfected with p.Gly45Glu under physiological Ca^2+^ concentrations die after two days of transfection (Stong et al., 2006). Conversely, the novel Cx26 p.Gly45Arg variant changes the nonpolar Gly amino acid to a positively charged, basic amino acid. Whereas protein localization and the formation of gap junctions remained unchanged, this substitution disrupted the permeation of negatively charged inositol 1,4,5-triphosphate (IP_3_) and altered cellular function. Cx26 p.Gly45Arg transfected cells, unlike cells transfected with p.Gly45Glu, were able to thrive at physiologic Ca^2+^ concentrations. In another variant involved in KID, p.Gly12Arg, a positively charged (basic) arginine replaces the nonpolar glycine amino acid, just as in the novel p.Gly45Arg variant. p.Gly12Arg affects connexon gating polarity and the regulation of ion permeation, similar to other dominant variants in this region (Purnick et al., 2000; Lazic et al., 2012). Although the p.Gly45Arg variant reflects the same amino acid substitution, it is situated at a different location (E1) in the protein and results in nonsyndromic hearing loss instead of KID.

The p.Gly45Glu and p.Gly45Arg variants in our study change the charge of the amino acid, thus producing substantially different effects on the structure and functionality of the Cx26 hemichannels. However, not all dominant variants result in a change of charge, polarity, or hydrophobicity. For example, the dominant negative variant p.Ala40Val replaces one nonpolar hydrophobic amino acid (alanine) with another (valine) and still has a profound effect on connexon functionality (Lai-Cheong, Arita & McGrath, 2007). p.Ala40Val and p.Asp50Asn are both examples of gain of function variants that produce KID manifestations by affecting calcium hemostasis, but they do so through different underlying mechanisms of calcium regulation. Both variants exhibit reduced inhibition by extracellular Ca^2+^, but p.Asp50Asn also shows reduced hemichannel permeability to Ca^2+^ (Lilly et al., 2016). Conversely, p.Gly45Glu displays a considerable increase in Ca^2+^ permeability (Sánchez et al., 2010). Ca^2+^ hemostasis seemed unaffected by the novel p.Gly45Arg variant, which is supported by the lack of epidermal pathology. Overall, these examples illustrate that the functional effects of Cx26 variants have a range of underlying mechanisms, including polarity, charge, changes in Ca^2+^ hemostasis, and variant location in the protein.

Most dominant amino acid substitutions in the N-terminus and E1 regions of Cx26 are associated with a syndromic phenotype (http://davinci.crg.es/deafness/). In this study we have described the novel variant p.Gly45Arg and demonstrated that changing the highly conserved nonpolar amino acid at position 45 produces contrasting effects on the structure and functionality of the Cx26 protein when compared to p.Gly45Glu. These effects may depend on the charge of the substituted amino acid: a positive charge results in the nonsyndromic hearing loss phenotype associated with p.Gly45Arg whereas a negative charge produces the syndromic fatal KID syndrome observed with p.Gly45Glu.

##  Supplemental Information

10.7717/peerj.2494/supp-1 Data S1 Raw data associated with Fig. 3

## Additional Information and Declarations

### Data Availability

The following information was supplied regarding data availability: Raw data associated with Fig. 3 has been supplied as Data S1.
